# Advancements in pseudouridine modifying enzyme and cancer

**DOI:** 10.3389/fcell.2024.1465546

**Published:** 2024-12-16

**Authors:** Kaijie Liu, Shujun Zhang, Yafeng Liu, Xinjun Hu, Xinyu Gu

**Affiliations:** ^1^ Department of Infectious Diseases, The First Affiliated Hospital, College of Clinical Medicine, Henan University of Science and Technology, Luoyang, Henan, China; ^2^ Henan Medical Key Laboratory of Gastrointestinal Microecology and Hepatology, Luoyang, China; ^3^ Department of Oncology, The First Affiliated Hospital, College of Clinical Medicine, Henan University of Science and Technology, Luoyang, Henan, China

**Keywords:** epigenetics, pseudouridine, RNA, pseudouridine-modifying enzyme, tumor

## Abstract

Pseudouridine (Ψ) is a post-transcriptional modifier of RNA, often referred to as the ‘fifth nucleotide’ owing to its regulatory role in various biological functions as well as because of its significant involvement in the pathogenesis of human cancer. In recent years, research has revealed various Ψ modifications in different RNA types, including messenger RNA, transfer RNA, ribosomal RNA, small nuclear RNA, and long noncoding RNA. Pseudouridylation can significantly alter RNA structure and thermodynamic stability, as the Ψ-adenine (A) base pair is more stable than the typical uridine (U)-A base pair is due to its structural similarity to adenine. Studies have linked Ψ expression to the development and progression of several digestive system cancers, such as liver cancer and colorectal cancer, and nondigestive system cancers, such as breast cancer, non-small cell lung cancer, prostate cancer, glioblastoma, ovarian cancer, oral squamous cell carcinoma, and pituitary cancer. The present review briefly outlines the chemical structure, synthesis, and regulatory mechanisms of Ψ. This review summarizes the effects of pseudouridylation on various substrates of RNA and briefly discusses methods for detecting Ψ. Last, it focuses on how RNA pseudouridylation influences different cancers, emphasizing the search for novel approaches to cancer diagnosis, treatment, and prognosis through Ψ modification.

## 1 Introduction

Pseudouridine (Ψ) is a post-transcriptional modification of RNA that plays a role in epigenetics. Epigenetics involves heritable changes in gene function without DNA sequence alternations, ultimately leading to observable changes in phenotype (Gayon, 2016). Epigenetic modifications can alter DNA, proteins, and RNA through chemical modifications, influencing gene expression. These modifications can affect various levels of gene regulation, including transcription, splicing, stability, translation, nucleosome assembly, and chromatin structure, impacting both physiological and pathological processes in cells, as well as the traits of offspring. Common epigenetic modifications include DNA methylation, histone modifications, RNA modifications, and chromatin remodeling (Li, 2021). Recent advances in detection technologies have highlighted the diversity of RNA and increased our understanding of the role of RNA modifications in epigenetics.

**FIGURE 1:**
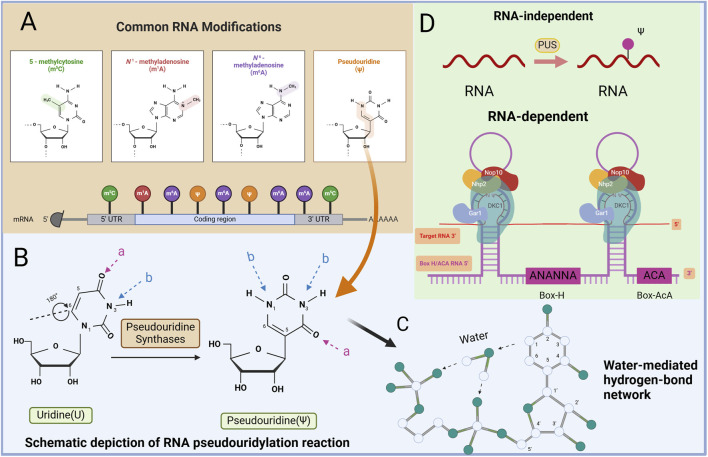
Mapping of RNA modifications and RNA pseudouridylation machinery. **(A)** There are many ways that RNA can be modified, such as N6-methyladenine (m6A), N1-methyladenine (m1A), 5-hydroxymethylcytosine (hm5C), Ψ, etc. **(B)** Schematic depiction of RNA pseudouridylation reaction catalyzed by PUS. PUS mediate the isomerization of U to ψ, which results in an extra hydrogen bond donor **(B)** and the same number of hydrogen bond acceptors **(A)**. **(C)** Water-mediated hydrogen-bond network. Schematic depiction of the Ψ-specific, water-mediated hydrogen-bond network. The water molecule and positions of the base and the sugar are indicated. **(D)** The mechanism of Ψ regulation. There are two main ways of pseudouracilylation of RNA substrates in eukaryotes.

RNA modification involves altering the chemical properties and structure of RNA through the addition of chemical groups, which in turn regulate RNA stability, transport, translation, and regulation (Karthiya et al., 2020; Zhao et al., 2016; Nachtergaele and He, 2018). Various modifications can occur, including N6-methyladenine, N1-methyladenine, 5-hydroxymethylcytosine, and Ψ (Figure 1A). These modifications impact RNA properties such as stability, translation efficiency, and recognition, ultimately affecting gene expression. The role of RNA modification, particularly in human diseases such as cancer, has become increasingly significant and is a major focus of current biological research (Nachtergaele and He, 2018). Over 100 post-transcriptional modifications have been identified in RNA, with increasing evidence indicating their critical role in epigenetic gene expression regulation in both normal physiology and disease (Nachtergaele and He, 2018; Machnicka et al., 2012). Among them, Ψ is the most prevalent RNA modification known to influence RNA structure and function (Zhang et al., 2019; Hamma et al., 2006).

Recent research has revealed Ψ modifications in various RNA types, including messenger RNA (mRNA), transfer RNA (tRNA, tsRNA), ribosomal RNA (rRNA), small nuclear RNA (snRNA), and long noncoding RNA (lncRNA) (Schwartz et al., 2014). This modification, characterized by a geometrically similar Watson–Crick base, enhances the thermodynamic stability of RNA compared with the usual uridine (U)-adenine (A) base pair. However, abnormal pseudouridylation is implicated in numerous human diseases, particularly cancer. Studies have linked Ψ to various cancers, such as liver cancer (Galardy et al., 2012; Lan et al., 2023) and gastric cancer (GC) (Chang et al., 2024), colorectal cancer (CRC) (Kan et al., 2021; Hou et al., 2019; Zhang Q. et al., 2023; Du et al., 2022; Song et al., 2021), breast cancer (BC) (Montanaro et al., 2006; Gu et al., 2008; Zhao et al., 2004), non-small cell lung cancer (NSCLC) (Penzo et al., 2015; Liu et al., 2021; Ji et al., 2020; Zhang G. et al., 2023), prostate cancer (PC) (Sieron et al., 2009; Jana et al., 2017), glioblastoma (GBM) (Miao et al., 2019; Cui et al., 2021; Ding et al., 2024), ovarian cancer (OC) (Li et al., 2021), oral squamous cell carcinoma (OSCC) (Alawi et al., 2010; Sridharan et al., 2019), and pituitary cancer (Bellodi et al., 2010). Ψ modifications are closely associated with apoptosis, metabolism, and cell cycle regulation in cancer cells. Furthermore, these modifications significantly influence cancer diagnosis, prognosis, and treatment strategies (Xue et al., 2022).

In the present review, we briefly introduce the chemical structure, synthesis, and regulation of Ψ, summarize the effects of pseudouridylation on various RNA molecules, and outline methods for detecting Ψ. In addition, we focus on the impact of RNA pseudouridylation on different cancers, emphasizing the search for new methods for cancer diagnosis, treatment, and prognosis prediction through Ψ modification.

## 2 Structure of pseudouridine

Ψ is a well-studied derivative of U found in all RNA categories (Schwartz et al., 2014). It is formed from U through a post-transcriptional isomerization reaction, resulting in the development of a C-C glycoside isomer. This modification incorporates the C5 atom of the nucleobase into the glycosidic bond, introducing additional hydrogen bond donors to the non-Watson–Crick edges of Ψ (Figure 1B) (Borchardt et al., 2020). Essentially, U isomerizes by a 180-degree rotation along the C6‒N3 axis, leading to unusual C‒C bond formation between the base and sugar at the C1′ and C5 positions in Ψ, unlike the normal N‒C bond (Charette and Gray, 2008). Compared with U, Ψ has a more flexible conformation, and its free N1-H group serves as a hydrogen bond donor, facilitating acyl transfer and novel pairing interactions (Lane et al., 1995; Lane et al., 2002). The hydrogen bond between water molecules O and N1-H in Ψ is stronger than that between water molecules C5-H, enhancing localized RNA packing and stability. This reduction in flexibility results in a rigid phosphodiester backbone, which is crucial for stabilizing the RNA structure through Ψ modification (Figure 1C) (Davis et al., 1998; Davis, 1995; Durant and Davis, 1999).

## 3 Synthesis and regulation of Ψ

### 3.1 Synthesis of pseudouridine

The formation of Ψs in RNA occurs through two main mechanisms. One mechanism involves the action of pseudouridine synthase (PUS) proteins, which convert U to Ψ. In this mechanism, the protein recognizes the target site and catalyzes the conversion. The second mechanism involves a complex formed by a type of H/ACA box snRNA and its corresponding protein. In this mechanism, the RNA serves a recognition role, guiding the protein to the target site while the protein catalyzes the conversion to Ψ (Carlile et al., 2019; Safra et al., 2017). These mechanisms highlight both RNA-independent and RNA-dependent pathways for pseudouridylation (Zhao et al., 2018).

In an RNA-independent mechanism, PUS can directly catalyze the conversion of U to Ψ in RNA substrates. These enzymes possess both substrate recognition and transglycosylation capabilities and operate without the need for auxiliary factors (Figure 1D). Among the 13 human PUSs, 12 are “stand-alone” enzymes whose RNA substrates are primarily identified on the basis of sequence or structural features without assistance from helper RNAs (Carlile et al., 2019; Safra et al., 2017).

The RNA-dependent mechanism involves catalysis through H/ACA ribonucleoprotein (RNP) complexes (Karijolich and Yu, 2011). Each RNP contains a unique cassette H/ACA small RNA molecule and four evolutionarily conserved core proteins (Karijolich and Yu, 2011). H/ACA RNA is highly conserved among small noncoding RNAs and serves to guide the pseudouridylation of various RNA species. The core proteins of H/ACA RNPs, including dyskerin (DKC1), nonhistone protein 2, glycine arginine-rich protein 1, and nucleolar protein 10, play crucial roles in substrate recognition and base pairing of the substrate RNA. Box H/ACA RNAs have a median length of 133 nucleotides and exhibit a hairpin-hinge-hairpin-tail secondary structure. Within the internal hinge region, an H-box motif (ANANNA) is present, whereas near the 3′end, an ACA box motif (ACA) can be found. Each hairpin structure contains a pseudouridylation pocket that facilitates target RNA recognition through complementary base-pairing interactions. H/ACA RNAs can possess functional pseudouridylation pockets in both of their hairpins, enabling them to direct the pseudouridylation of uridines in separate RNAs or to target separate uridines within the same RNA. Among these proteins, DKC1 exhibits catalytic activity, enabling the direct pseudouridylation of RNA guided by H/ACA RNA molecules (Sánchez-Vásquez et al., 2018).

### 3.2 Regulation of pseudouridine

In humans, Ψ is primarily formed by enzymes belonging to the PUS family, specifically PUS1 to PUS10. These enzymes, known as Ψ “writers,” catalyze the isomerization of U to Ψ (Xue et al., 2022). On the basis of sequence homology, these enzymes can be classified into six families: RluA, RsuA, TruA, TruB, TruD, and the PUS10 family, named after their bacterial counterparts (Borchardt et al., 2020). For example, the TruA family of proteins catalyzes the conversion of U to Ψ at positions 38 to 40 in the anticodon loop of tRNA, the TruB family acts at position 55 of the TΨC loop of tRNA, and the TruD family modifies at position 15 in the D stem loop of tRNA. The RsuA family of proteins exhibits highly site-specific catalytic activity on rRNA sites in prokaryotes. Moreover, the RluA family primarily catalyzes Ψ modifications on rRNA in prokaryotes. However, compared with the RsuA family of proteins, the RluA family of proteins has not only different catalytic sites but also a broader catalytic site area (Borchardt et al., 2020).

In addition to its primary enzymatic activity, pseudouridine synthase also serves a distinct function. For example, DKC1, an evolutionarily conserved protein, is a component of the human telomerase complex (Qin et al., 2024). It participates in and stabilizes human telomerase RNA (hTR), thereby contributing to the protection of telomere integrity. The pseudouridine synthase TruB serves as a companion to tRNA. In addition to its modification activity, TruB’s capacity to fold tRNAs is essential for cellular adaptation. TruB interacts with both misfolded and properly folded tRNAs, providing misfolded tRNAs with a second opportunity to achieve correct folding (Keffer-Wilkes et al., 2016). The human pseudouridine synthases TruB1 and PUS10 also play pivotal roles in microRNA biosynthesis. Let-7, one of the first microRNAs (miRNAs) identified, promotes differentiation during development and functions as a tumor suppressor in various cancers. A recent report indicated that the human pseudouridine synthase TruB1 enhances the maturation of let-7 by directly binding to pri-let-7 and recruiting the Drosha-DGCR8 microprocessor complex (Xuan et al., 2024). Prior studies have demonstrated that depletion of PUS10 results in a significant reduction in the expression levels of numerous mature miRNAs, accompanied by the accumulation of unprocessed primary microRNAs (PRI-miRNAs) in various human cell types. Mechanistically, PUS10 directly interacts with pri-miRNAs and collaborates with microprocessors to facilitate miRNA biogenesis (Song et al., 2020).

Currently, the “reader and eraser” proteins for Ψ modification remain uncertain. This uncertainty could be attributed to the inert nature of the C‒C bond formed between the base and ribose in Ψ, which is much more stable than the C‒N bond. This stability suggests that the pseudouridylation process is likely irreversible (Xue et al., 2022).

## 4 Effect of pseudouridylation on RNA molecules

Ψ is a prevalent RNA modification and a key mechanism of post-transcriptional modification. In recent years, research on RNA Ψ modifications has expanded to include various RNA types, such as mRNAs, tRNAs, tsRNAs, rRNAs, snRNAs, and miRNAs (Schwartz et al., 2014). This modification can significantly impact RNA structure and function (summarized in Table 1). Compared with the usual U-A base pair, RNA pseudouridylation can profoundly affect RNA structure and thermodynamic stability due to the more stable base pairing of Ψ with a Watson–Crick base (Davis, 1995; Kierzek et al., 2014).

**TABLE 1:** The effect of pseudouridylation on RNA molecules.

name	Influence	References
mRNA	mRNA stability, half-life, mRNA coding and pre-mRNA splicing	(Li X. et al., 2015, Karijolich and Yu, 2011, Anderson et al., 2010, Karikó et al., 2012, Martinez and Gilbert, 2018,Karikó et al., 2008)
tRNA	Stabilize spatial structure and improve translation efficiency and accuracy	(Uddin et al., 2020, Lorenz et al., 2017)
tsRNA	Promote the synthesis of tsRNA	(Li et al., 2018)
rRNA	Protein translation and ribosomal biogenesis	(Jiang et al., 2015, Garus and Autexier, 2021)
snRNA	snRNA biogenesis, spliceosome assembly, and splicing	(R, 1988, Wu et al., 2011)

Pseudouridylation plays a crucial role in the molecular and biological functions of both noncoding and coding RNAs. Here, we provide a summary and discussion of several main categories of RNAs affected by pseudouridylation.

### 4.1 Effects of pseudouridylation on messenger RNA

**FIGURE 2:**
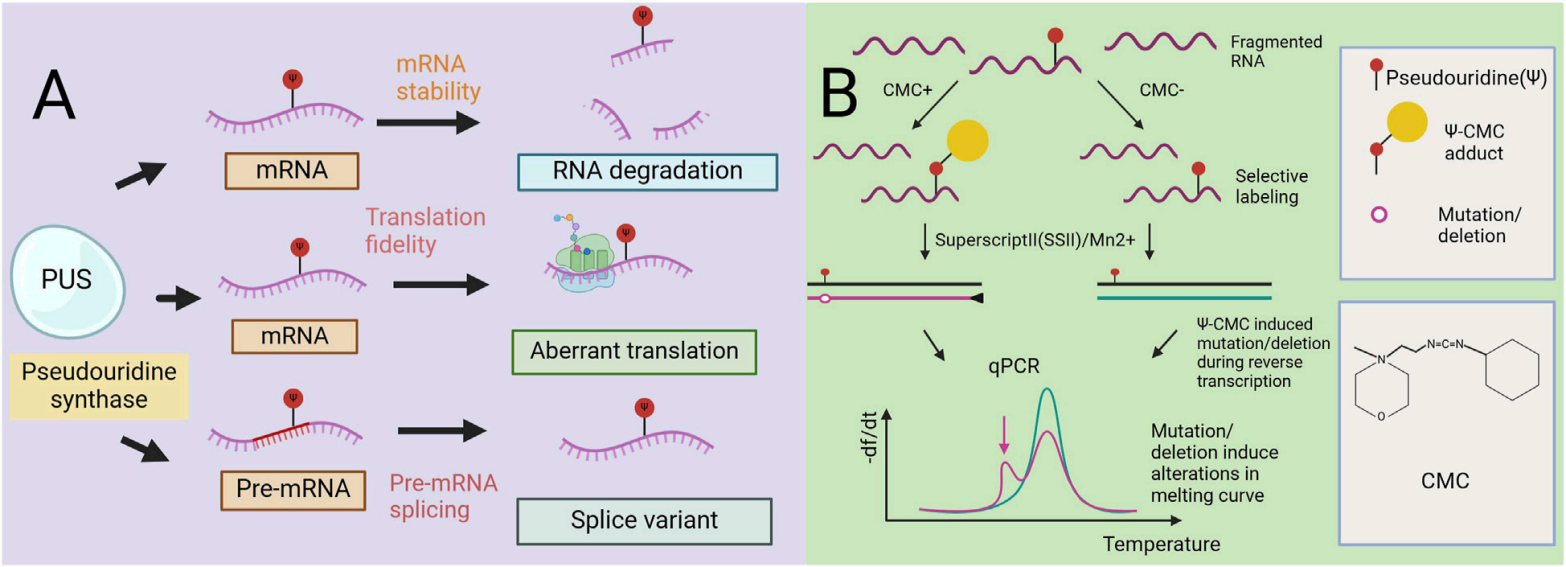
The role of pseudouracil in mRNA metabolism. Mechanism diagram of a Radiolabeling-Free, qPCR-Based Method for Locus-Specific Pseudouridine Detection. **(A)** Pseudouridine (Ψ) impacts multiple facets of mRNA metabolism such as pre-mRNA splicing, RNA stability, and translation. Ψ can affect the local mRNA structure of sub-strate RNA and thereby regulate its mRNA stability. Very often, pseudouridylated mRNA codons are associated with unusual base pairing, which alters the fidelity of translation. Further, pre-mRNA pseudouridylation can affect the splicing machinery and thus regulate alternative splicing. **(B)** Workflow of the method (qPCR-Based). Y-containing RNA is specifically labeled by CMC, and then reverse transcribedbySuperscriptII(SSII) with Mn2+ buffer.The Y–CMC adducts cause SSII to introduce amutation/deletion at or around the Y site in the synthesized cDNA, thus giving rise to anew peak (indicated by the red arrow) in the melting curve of qPCR products.

Initially, discovered primarily in noncoding RNAs such as tRNA and rRNA, Ψ has garnered attention in mRNA research over the past decade. Both *in vivo* and *in vitro* studies have demonstrated that Ψ plays a role in mRNA stability, with Ψ-containing mRNAs being more stable. Ψ can impact various aspects of mRNA metabolism, including pre-mRNA splicing, RNA stability, and translation (Figure 2A). By affecting the local mRNA structure, Ψ can regulate the stability and half-life of its mRNA substrates. Furthermore, pseudouridylated mRNA codons can pair with unusual bases, potentially altering translation fidelity.

The ability of pseudouridine to modify base-pairing interactions enables it to affect not only RNA structure but also mRNA coding, highlighting its potential as a regulatory element (Li X. et al., 2015). Research has demonstrated that the incorporation of pseudouridine modifications in the stop codon can convert it into a functional codon. These findings indicate that pseudouridine modifications in the stop codon can significantly alter its coding ability (Karijolich and Yu, 2011). Additionally, other researchers have reported that when mRNAs containing pseudouridine modifications are transcribed *in vitro* and subsequently injected into the body, both the translation rate and stability of the modified RNA are greater than those of mRNAs without such modifications (Anderson et al., 2010; Karikó et al., 2012). Interestingly, another research team discovered that pseudouridine is enriched in the pre-mRNA selective splicing region and near the splicing site. They reported that a single endogenous pseudouridine in pre-mRNA exerts a direct biochemical effect on pre-mRNA splicing *in vitro*. To identify which enzymes target precursor mRNAs and may regulate splicing, an *in vitro* research team identified PUS1, PUS7, and RPUSD4 as precursor mRNA pseudouridylases. Mechanistically, pseudouridine in precursor mRNAs can influence splicing through three primary mechanisms: altering the precursor mRNA–SNRNA interaction, regulating the precursor mRNA–protein interaction, or affecting the precursor mRNA secondary structure (Martinez and Gilbert, 2018). In summary, the results of this study suggest that pseudouridine and pre-mRNA-modified pseudouridine synthase are novel regulators of pre-mRNA processing. Pre-mRNA pseudouridylation can influence the splicing mechanism, thereby modulating alternative splicing (Karikó et al., 2008).

### 4.2 Effects of pseudouridylation on RNA transfer

Several studies have confirmed the presence of numerous pseudouridylation sites in tRNAs. Notably, Ψ can be found not only in the stem and loop of the tRNA anticodon but also in the TΨC loop and D stem. This modification plays a key role in stabilizing the spatial structure of tRNA and facilitating the recognition and pairing of codons and anticodons, thereby increasing translation efficiency and accuracy (Uddin et al., 2020). In addition, pseudouridylation is essential for the correct folding of tRNA and its efficient translation, serving as a recognition marker for aminoacyl-tRNA synthetase (Lorenz et al., 2017). Pseudouridylation potentially influences tRNA aminoacylation, although conclusive evidence is currently lacking. Moreover, tissue-specific pseudouridylation of tRNA might help fine-tune tRNA function to align with cell type-specific translational requirements (Brandmayr et al., 2012).

In addition, pseudouridylation has reportedly been implicated in the synthesis and function of tRNA-derived short RNAs that regulate translation (Li et al., 2018). For example, inactivation of PUS7 in embryonic stem cells disrupts tRNA-derived fragment (tRF)-mediated translational control, leading to increased protein biosynthesis and defects in germ layer specification. This mechanism could involve the stability of the cap-binding complex (Guzzi et al., 2018; Ivanov et al., 2011). Considering the diverse activities of tRNA-derived fragments and the limited understanding of their synthesis and regulation, other PUSs may also influence human gene expression through tsRNAs.

### 4.3 Effects of pseudouridylation on ribosomal RNA

Ψ is widely distributed in rRNA, existing in almost all rRNAs. Its function has been elucidated through the discovery of H/ACA RNA, which is responsible for rRNA pseudouridylation (Ganot et al., 1997). Ψ is distributed across various functional regions of rRNA, typically clustering in critical regions for protein translation and ribosomal biogenesis. These regions include the tRNA stem‒loop structure, mRNA interaction sites, decoding centers, peptidyltransferase centers, and sites of interaction between large and small ribosomal subunits, all of which are crucial for the biological function of rRNA (Jiang et al., 2015). DKC1 is the primary PUS responsible for mediating rRNA pseudouridylation and is therefore essential for ribosomal biogenesis (Garus and Autexier, 2021). DKC1 deficiency is associated with decreased translation fidelity and impaired translation initiation (Jack et al., 2011). Research has shown that DKC1 deficiency in human cells can lead to defects in rRNA uridine modification, ultimately altering ribosomal activity (Garus and Autexier, 2021).

### 4.4 Effects of pseudouridylation on small nuclear RNA

Research has revealed that all major spliceosome snRNAs undergo pseudouridylation, with U2 snRNAs showing the most widespread pseudouridylation. In vertebrates, U2 snRNAs carry 13 constitutive Ψ modifications, which are clustered mainly in functional regions crucial for U2 snRNP biogenesis, spliceosome assembly, and splicing (R, 1988). Moreover, under stress conditions such as nutritional deprivation and heat shock, yeast U2 snRNA can undergo inducible pseudouridylation at new sites. These inducible Ψ modifications also impact the splicing of precursor mRNAs (Wu et al., 2011).

### 4.5 Effects of pseudouridylation on long noncoding RNAs

Ψ is the most prevalent RNA modification found not only in rich noncoding RNAs such as rRNA, snRNA, and tRNA but also in other noncoding RNAs. For example, pseudouridylation of lncRNAs has been observed in numerous organisms, although the specific mechanisms are yet to be fully elucidated (Dinescu et al., 2019). The emergence of transcriptome-wide Ψ mapping technologies has led to the discovery of new Ψ sites in relatively low-abundance noncoding RNAs, presenting both a challenge and an opportunity to explore their mechanisms. Further research will continue to unveil the role and significance of Ψ in RNA.

## 5 Detection of pseudouridine

Several methods are available for detecting Ψ modifications in RNA. Here, we briefly introduce some of these methods and discuss their respective advantages and disadvantages (summarized in Table 2).

**TABLE 2:** Detection of pseudouridine.

Classifcation	name	Advantages	Disadvantages	References
Mass Spectrometry-Based Methods to Detect Pseudouridine	No data yet	Detailed at atomic resolution	Lack of high-throughput potential for large-scale research	(Addepalli and Limbach, 2011)
High-Throughput Sequencing-Based Methods to Detect Pseudouridine	RBS-Seq	Capable of identifying all known Ψ sites	cause base deletion	(Khoddami et al., 2019)
	HydraPsiSeq	Sensitive, reliable and quantitative	No data yet	(Marchand et al., 2020, Marchand et al., 2022)
	PRAISE			
	BID-seq	Strong quantitative ability and wide practicability	abundance low or multiple uridine adjacent	(Zhang M. et al., 2023, Dai et al., 2023, Zhang et al., 2024)
Quantitative detection of basic RT-PCR Ψ Method	RT-PCR	sensitive	For two or more closely spaced RNA Ψ Site, it can only accurately evaluate the 3′Ψ site	(Zhang et al., 2019)
Rapid detection of site specificity based on qPCR Ψ Method	qPCR	Low cost, no radioactive labeling, fast detection	Unable to quantify	(Lei and Yi, 2017)

### 5.1 Mass spectrometry-based methods

In recent years, mass spectrometry technology has made significant strides, aided by advancements in data analysis software. This technology has become a cornerstone in the study of biological macromolecules. Mass spectrometry is now the preferred method for measuring the Ψ abundance in RNA because of its ability to provide detailed atomic resolution. Typically, this method targets purified RNA species. However, since Ψ and U have the same mass, specific ions produced during fragmentation are used for detection (Addepalli and Limbach, 2011). This approach has been successfully utilized to determine the absolute Ψ level in rRNA from human TK6 cells (Taoka et al., 2018). In addition, it has been used to map the complete Ψ content in snRNAs from adult HEK293T cells, enabling analysis of the Ψ content in mRNAs from HEK293 cells (Yamauchi et al., 2016; Li XY. et al., 2015).

### 5.2 High-throughput sequencing-based methods

This method, previously known by various names, such as PseudoSq (Carlile et al., 2014) and Ψ-seq (Schwartz et al., 2014) (in yeast and mammalian cells), PSI-seq (Preiss et al., 2014) (yeast), and CeU-seq (Li XY. et al., 2015) (mammalian cells), operates via the same principle. It involves treating RNA with the chemical N-cycloxyl-N'-(2-morpholinoethyl)-carboximide (CMC), which adds a large group to Ψ. This modification halts reverse transcriptase during cDNA synthesis, allowing the identification of pseudouridylation sites across the entire transcriptome through RNA-seq. These CMC-based technologies have been effective in identifying Ψ loci, particularly highly abundant RNAs such as rRNA, and have even been used to identify candidates in mRNA Ψ loci (Zaringhalam and Papavasiliou, 2016). However, validating these sites poses a challenge. The method relies on selective chemical labeling with CMC, and validation requires a large amount of pure target RNA, which is often not feasible for most mRNAs. Consequently, previous studies have either lacked validation (Carlile et al., 2014) or validated only a small number (<5) of high-abundance candidate sites (Preiss et al., 2014).

In contrast, RNA bisulfite-seq (RBS-seq) offers a straightforward, well-suited, high-throughput validation approach for detecting mRNA pseudouridylation. In this method, the Ψ-dependent deletion feature in the corresponding cDNA is easily amplified via gene-specific PCR (polymerase chain reaction) amplification and quantified through high-throughput sequencing of barcode amplicons (Li XY. et al., 2015). Moreover, by creating a specialized pipeline for rRNA analysis, RBS-seq allows for the identification of nearly all known Ψ sites on rRNA. However, this method may result in the loss of one or two bases adjacent to the target Ψ (Khoddami et al., 2019).

Compared with previously described high-throughput methods, a unique feature of HydraPsiSeq is that it provides, for the first time, quantitative information about the amount of pseudouridylation at any particular position of an RNA molecule with nucleotide precision. This method is a sensitive, reliable and quantitative approach for profiling the pseudouridine of RNA on the basis of chemistry orthogonal to classical CMC derivatization. To achieve this goal, the researchers explored and optimized random RNA cleavage at uridine residues by hydrazine, followed by aniline treatment for RNA chain scission at abasic sites. Protected residues (negative hits) reveal the presence of pseudouridines on the basis of their resistance to hydrazine-dependent scission. These protection signals were compared to the efficiency of neighboring cleavages at unmodified uridines to obtain quantitative information on the pseudouridylation levels. HydraPsiSeq is a novel quantitative Ψ mapping technique that relies on specific protection from hydrazine/aniline cleavage. HydraPsiSeq is quantitative because the obtained signal directly reflects the pseudouridine level. HydraPsiSeq does not involve the selective enrichment of Ψ-modified RNAs, and as such, it is particularly efficient for abundant cellular RNAs, such as rRNAs and tRNAs. Furthermore, normalization to natural unmodified RNA and/or synthetic *in vitro* transcripts allows for absolute measurement of modification levels (Marchand et al., 2020; Marchand et al., 2022).

In addition to the aforementioned assays, recent studies have introduced novel quantitative sequencing methods, specifically PRAISE (Zhang M. et al., 2023) and BID-seq (Dai et al., 2023). These techniques utilize the bisulfite labeling reaction and Ψ-bisulfite adducts, which result in deletions during the reverse transcription (RT) process. Both methods have demonstrated their quantitative capabilities via the use of synthetic spike-in RNA and rRNA. The list of Ψ sites in mRNA was expanded through a stoichiometric approach. Additionally, the high reproducibility of the Ψ modification sites and stoichiometry across biological replicates was established. Their stability extends to various library constructs, thereby enhancing their applicability for widespread use in quantitative Ψ detection. These methods affirm their reliability and potential for broader application in Ψ research. However, challenges remain: PRAISE and BID-seq struggle to precisely define Ψ sites within constitutive ‘U' contexts, and the calling of Ψs in low-abundance transcripts may rely more heavily on sequencing depth. Validation of these modification sites may necessitate targeted sequencing. The detection of Ψ sites by BID-seq is based on the absence of a signature generated by Ψ-BS sites during the reverse transcription (RT) process. Adequate reading coverage may be essential for identifying low-level modification sites in low-level RNAs. When a Ψ site is adjacent to one or more uridines, determining the precise pseudouridylation site becomes challenging, as the Ψ will produce the same deletion pattern at any location (Zhang et al., 2024). Nevertheless, these studies offer new avenues for investigating the biological relevance and regulatory mechanisms of variable Ψ stoichiometry.

Recently, a research team developed a method known as bisulfite binding retarding junction (BIHIND), which is capable of detecting and quantifying pseudouridine (Ψ) sites on rRNA, mRNA, and noncoding RNA. BIHIND can be integrated with quantitative PCR (Bihind-QPCR) for the quantitative detection of the Ψ portion at a single modification site, or it can be combined with next-generation sequencing (Bihind-SEQ) for high-throughput sequencing of Ψ without the necessity for reverse transcription. We have incorporated the relevant content and cited the appropriate references in the paper (Zhao et al., 2024).

### 5.3 CLAP

In recent years, a quantitative method based on RT‒PCR (reverse transcription‒polymerase chain reaction) called CLAP (CMC‒RT and linker‒assisted PCR) has been developed to detect and quantify mRNA/lncRNA Ψ sites. This method is highly sensitive, requiring only a small amount of RNA. It relies on the CMC-induced pause of reverse transcriptase at Ψ sites, followed by site-specific ligation and PCR. CLAP generates two different PCR products in the same sample, corresponding to the modified and unmodified sites, which can be visualized via gel electrophoresis. However, for two or more closely spaced RNA Ψ sites, CLAP may only accurately evaluate the 3′Ψ location (Zhang et al., 2019).

### 5.4 A radiolabeling-free, qPCR-based method for locus-specific pseudouridine detection

Currently, high-throughput methods for detecting Ψ, while effective, can be costly and require complex bioinformatics analysis. Although methods have been developed for locus-specific Ψ detection, they often involve radioactive labeling of RNA, necessitate advanced experimental skills, and can be time-consuming.

Here, we introduce a nonradioactive labeled, quantitative polymerase chain reaction (qPCR)-based rapid detection method for site-specific Ψ detection (Lei and Yi, 2017). This method relies on Ψ-induced mutations/deletions during cDNA synthesis with CMC, leading to the generation of qPCR products with different melting temperatures. Unlike existing methods that rely on RT stopping caused by Ψ-CMC, this method utilizes new RT conditions that allow read-through of Ψ-CMC, resulting in mutations and/or deletions in cDNA. These changes alter the melting curve of the qPCR products, enabling site-specific Ψ modification detection (Figure 2B) (Lei and Yi, 2017). In rRNA, this method detects individual and adjacent sites. In contrast, previous RT-dependent stopping methods are limited to detecting the RT site at the 3′end of RNA, restricting the detection of adjacent Ψ sites. Studies using this method designed primers covering Ψ1045 and Ψ1056 in 18S rRNA, observing an additional peak in the melting curve compared with that of Ψ1045 alone. Further high-throughput sequencing of the qPCR amplicons confirmed the detection of both Ψ sites. When applied to mRNA and lncRNA sites, the method resulted in less pronounced changes, likely due to the lower modification levels at these sites. However, when sufficient mRNA is present to produce a reliable melting curve, observable shifts in the curve are still detected. Overall, this qPCR-based method is highly sensitive for detecting Ψ, cost-effective, nonradioactive, and rapid, requires only commercial reagents for detecting Ψ and qPCR primers, and can be completed within 1.5 days. In addition, it effectively detects both abundant rRNA and low-abundance mRNAs. However, it does not provide quantitative determination of Ψ (Lei and Yi, 2017).

### 5.5 Nanopore sequencing

The concept of nanopore sequencing emerged in the 1980s and was made possible by a series of technological advances in nanopores and related motor proteins (Deamer et al., 2016). The technology relies on a nanoscale protein pore, or “nanopore,” that serves as a biosensor and is embedded in an electrically resistant polymer membrane (van Dijk et al., 2018). In an electrolytic solution, a constant voltage is applied to generate an ionic current through the nanopore so that negatively charged single-stranded DNA or RNA molecules are driven through the nanopore from the negatively charged “cis” side to the positively charged ‘trans’ side. The translocation speed is controlled by a motor protein that adds nucleic acid molecules through the nanopore in a stepwise manner. Changes in ionic current during translocation correspond to the nucleotide sequence present in the sensing region and are decoded via computational algorithms, enabling real-time sequencing of single molecules (Wang et al., 2021).

Separately, direct RNA sequencing via nanopore sequencing is a single-molecule method that directly analyzes RNA strands isolated from cells after end-ligation of adaptor sequences (Burrows and Fleming, 2023). Using this method, we can directly perform RNA sequencing on Ψs by monitoring the relationship between current and time. By combining the electrical current and base-calling data from the nanopore with dwell-time analysis from the helicase employed to deliver RNA to the nanopore, we were able to map the Ψ sites in nearly all sequence contexts (Burrows and Fleming, 2023).

Despite these advances, nanopore sequencing encounters limitations in the accurate and quantitative detection of Ψ, particularly owing to a low signal‒to‒noise ratio, especially in clustered Ψ sites such as rRNA, which can result in false positives. To address these challenges, alternative nanopore sequencing strategies incorporate chemical tools to selectively modify RNA modifications and enhance nanopore characteristics. For example, bisulfite-assisted nanopore sequencing is employed to obtain reliable base recognition error curves (Fleming et al., 2023). Additionally, nanopore-based direct RNA sequencing has emerged as a promising technology capable of detecting and quantifying multiple RNA modifications in natural RNA molecules. Similar assays have also been utilized to identify interferon-induced Ψ modifications in human mRNA (Huang et al., 2021).

## 6 Pseudouridine-modifying enzymes and cancer

**FIGURE 3:**
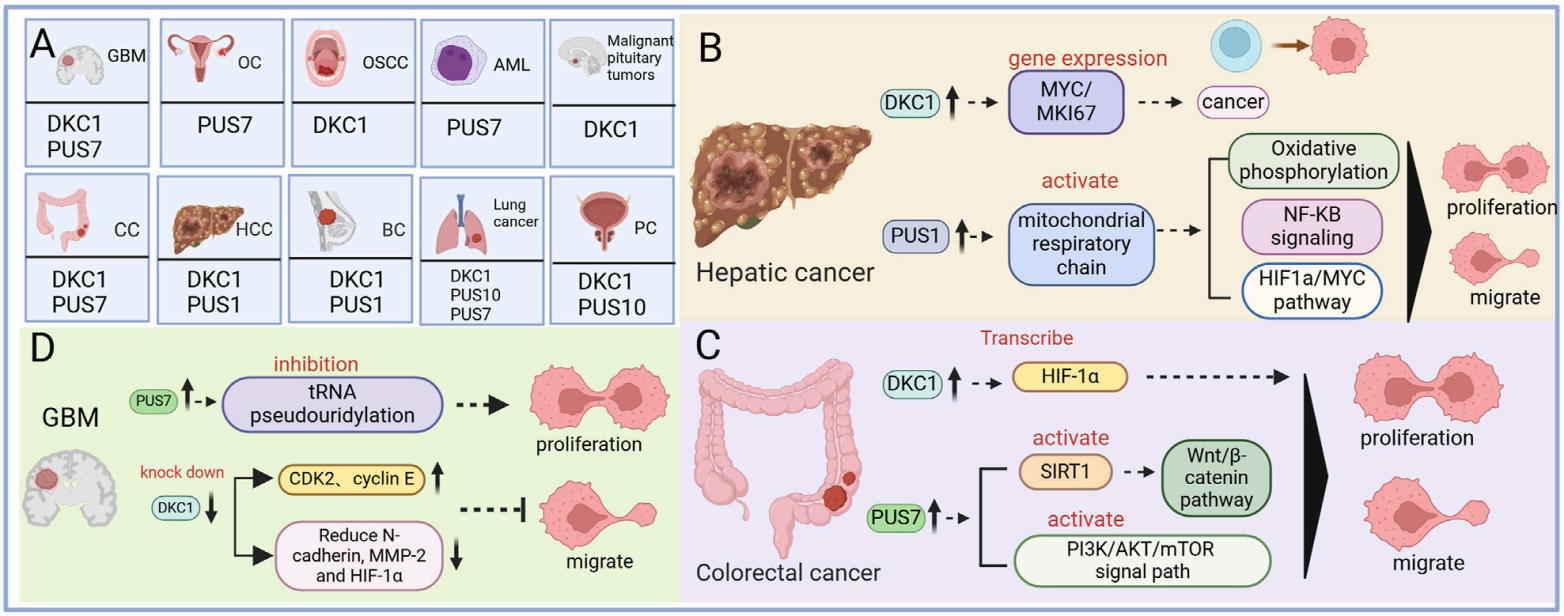
The mechanism of pseudouracil modification enzyme and cancer. **(A)** Diagram summarizing cancers covered in the review and their associated pseudouridine regulatory factor. **(B)** The regulatory mechanism of pseudouridine in Hepatic cancer. **(C)** The regulatory mechanism of pseudouridine in Colorectal cancer. **(D)** The regulatory mechanism of pseudouridine in Glioblastoma.

Cancer poses a significant threat to global human health (Sun et al., 2020), with incidence and mortality rates rising over the past 2 decades (Chen et al., 2021). RNA modification has emerged as a key player in cell proliferation, metabolism, and apoptosis and has potential in disease diagnosis, treatment, and prognosis. Among the RNA modifications implicated in human diseases, including cancer, N6-methyladenosine, 5-methylcytosine, and Ψ are the most notable (Xue et al., 2022). Numerous studies have highlighted the involvement of Ψ in various cancers, influencing their development, treatment, and prognosis. This section provides a summary of the role of Ψ in cancer, including digestive system tumors such as liver cancer and CRC, as well as nondigestive system tumors such as BC, NSCLC, PC, GBM, OC, OSCC, and pituitary cancer (Figure 3A). This information is presented in a tabulated format (summarized in Table 3).

**TABLE 3:** Pseudouridine and cancer.

Tumor	Factor	Gene description	Mechanism/Pathway	Expression	Biological function	Prognostic implication of Ψ regulators overexpression	References
Hepatocellular carcinoma	DKC1	Dyskerin pseudouridine synthase 1	MYC and MKI67 expression related	Elevated	Promote tumor cell survival by reducing translation efficiency and increasing translation error coding in HCC cells	Poor	(Galardy et al., 2012)
	PUS1	Pseudouridylate Synthase 1	NF-KB signal transduction, HIF1a pathway, MYC pathway	Elevated	Promote the migration, proliferation, and invasion of HCC cells	No data yet	(Lan et al., 2023)
Gastric Cancer	PUS7	Pseudouridylate Synthase 7	Pseudouridylation of ALKBH3mRNA	Downregulated	Gastric tumorigenesis	No data yet	(Chang et al., 2024)
Colorectal cancer	DKC1	Dyskerin pseudouridine synthase 1	Direct activation of HIF-1 α and RAS/RAF/MEK/ERK pathway	Elevated	Enhancing ribosomal protein expression to promote cancer progression	Poor	(Kan et al., 2021, Hou et al., 2019)
	PUS7	Pseudouridylate Synthase 7	activation Wnt/β-catenin、PI3K/AKT/mTOR pathway and HSP90/PUS7/LASP1	Elevated	Improving the proliferation and invasion of tumor cells	Poor	(Zhang Q. et al., 2023, Du et al., 2022, Song et al., 2021)
Breast cancer	DKC1	Dyskerin pseudouridine synthase 1	Low expression of DKC1 reduces telomerase activity	Downregulated	No data yet	No data yet	(Montanaro et al., 2006; Gu et al., 2008)
	PUS1	Pseudouridylate Synthase 1	Acting through pseudo uridylation of another co activator, steroid receptor RNA activator (SRA)	Elevated	No data yet	No data yet	(Zhao et al., 2004)
Non-small cell lung cancer	DKC1	Dyskerin pseudouridine synthase 1	Synergistic effect of VEGF/AKT/Bcl2/Caspase9 pathway with PCAT1	No data yet	Promote proliferation and migration, inhibit cell apoptosis	Poor	(Penzo et al., 2015, Liu et al., 2021)
	PUS10	Pseudouridylate Synthase 10	Rs9309336 may interfere with PUS10 expression and reduce tumor cell sensitivity to TRAIL	Elevated	Promoting the immortalization of tumor cells	No data yet	(Ji et al., 2020)
	PUS7	Pseudouridylate Synthase 7	No data yet	Elevated	Promote cell proliferation, migration and invasion	Poor	(Zhang G. et al., 2023)
Prostate cancer	DKC1	Dyskerin pseudouridine synthase 1	Related to the expression of hTR and MKI67	Elevated	Promote cell proliferation without any impact on cell apoptosis or aging	No data yet	(Sieron et al., 2009)
	PUS10	Pseudouridylate Synthase 10	Apoptosis of prostate cancer cells is affected by tumor necrosis factor-related mechanism	Elevated	Inducing TRAIL induced cell apoptosis	No data yet	(Jana et al., 2017)
Glioblastoma	DKC1	Dyskerin pseudouridine synthase 1	No data yet	Elevated	Promote tumor spread, invasion, and migration	Poor	(Miao et al., 2019)
	PUS7	Pseudouridylate Synthase 7	promotes tRNA pseudouridylation	Elevated	Promoting cell growth and self-renewal	Poor	(Cui et al., 2021, Ding et al., 2024)
Ovarian Cancer	PUS7	Pseudouridylate Synthase 7	Inducing DKC1 to induce pseudouridylation of RAP1B mRNA to specifically increase RAP1B protein levels	Elevated	Promote cell proliferation and migration	No data yet	(Li et al., 2021)
Oral squamous cell carcinomas	DKC1	Dyskerin pseudouridine synthase 1	No data yet	Elevated	Related to proliferation of active cells	No data yet	(Alawi et al., 2010, Sridharan et al., 2019)
AML	PUS7	Pseudouridylate Synthase 7	Enhance protein synthesis, thereby damaging stem cell differentiation	Missing	Promote the occurrence of leukemia	No data yet	(Guzzi et al., 2018)
Malignant pituitary tumors	DKC1	Dyskerin pseudouridine synthase 1	Reduce pituitary translation mediated by p27IRES	Downregulated	Promote the occurrence of tumors	No data yet	(Bellodi et al., 2010)

### 6.1 Hepatocellular carcinoma

Hepatocellular carcinoma (HCC) is the most prevalent form of liver cancer, constituting approximately 90% of all liver cancer cases. A study reported that serum Ψ levels are significantly elevated in patients with HCC compared with normal individuals, suggesting its potential as a valuable biochemical marker for HCC diagnosis. Studies have also revealed the upregulation of DKC1 in patients with HCC (Amuro et al., 1988). DKC1 plays a role in rRNA pseudouridylation and the stabilization of telomerase RNA (TERC). Its upregulation in HCC is associated with the expression of the MYC and MKI67 genes, implicating DKC1 in tumorigenesis (Galardy et al., 2012). Furthermore, high Ψ expression in HCC is correlated with poor prognosis (Galardy et al., 2012). Moreover, PUS upregulation has been observed in HCC, contributing to tumor progression. PUS1 upregulation leads to abnormal activation of the mitochondrial respiratory chain, promoting the migration, proliferation, and invasion of HCC cells through oxidative phosphorylation, NF-κB signal transduction, the HIF-1α pathway, and the MYC pathway (Figure 3B) (Lan et al., 2023).

### 6.2 Gastric cancer

Gastric cancer (GC) is the fifth most prevalent malignancy globally and ranks as the fourth leading cause of cancer-related mortality, particularly with a high incidence in East Asia (Thrift et al., 2023). Unfortunately, GC is frequently diagnosed at an advanced stage (Ishimoto et al., 2017), resulting in a poor prognosis for patients due to the lack of effective treatment options (Digklia and Wagner, 2016). Consequently, finding ways to inhibit the proliferation of gastric cancer cells and tumor growth has become a critical challenge. Recent findings indicate that PUS7 is significantly downregulated in gastric cancer tissue compared with adjacent nontumor tissue. Functional analyses demonstrated that PUS7 inhibits gastric cancer cell proliferation and tumor growth through its catalytic activity. Furthermore, PUS7 attenuates tumor growth by modifying the U696 site with pseudouridine, thereby increasing the translation efficiency of the ALKBH3 mRNA. Notably, ALKBH3 functions as a tumor suppressor in gastric cancer, and its expression is closely associated with PUS7 levels in tumor tissues. PUS7 enhances the translation efficiency of ALKBH3 via its ability to pseudouridylate ALKBH3 mRNA, consequently inhibiting gastric tumorigenesis. The expression levels of PUS7 and ALKBH3 are significantly correlated in gastric tumors, positioning them as potential prognostic indicators and therapeutic targets for patients with gastric cancer (Chang et al., 2024).

### 6.3 Colorectal cancer

According to the 2017 cancer statistics report from the American Cancer Society, CRC is among the top four cancers leading to death (Senerchia et al., 2016). Several studies have revealed that in CRC cells, DKC1 is highly expressed and binds to numerous ribosomal proteins, stabilizing their mRNAs (Kan et al., 2021; Turano et al., 2008). These ribosomal proteins then interact with HRAS, leading to downstream RAS/RAF/MEK/ERK pathway inhibition13. Increased DKC1 expression in patients with CRC has been associated with poor overall survival and progression-free survival, suggesting that DKC1 is a promising therapeutic target for CRC treatment (Kan et al., 2021). In addition, DKC1 can promote angiogenesis and metastasis in CRC by directly activating HIF-1α transcription. Elevated DKC1 expression is correlated with advanced TNM (primary tumor local lymph node distant metastasis) stage, lymph node metastasis, and poor prognosis in patients with CRC (Hou et al., 2019). Furthermore, PUS upregulation has been observed in CRC, contributing to tumor progression. PUS7 upregulation stabilizes the Wnt/β-catenin pathway by activating SIRT1, promoting CRC cell proliferation and activation (Zhang Q. et al., 2023). In addition, PUS7 enhances CRC proliferation and invasion by activating the PI3K/AKT/mTOR signaling pathway (Figure 3C) (Du et al., 2022).

Several researchers have reported the overexpression of PUS7 in colorectal cancer (CRC) tissues, which is correlated with advanced clinical stages and reduced overall survival rates. PUS7 silencing effectively repressed metastasis in colorectal cancer (CRC) cells, whereas PUS7 upregulation promoted metastasis independent of the catalytic activity of PUS7. LASP1 was identified as a downstream effector of PUS7, with forced expression of LASP1 negating the metastasis suppression induced by PUS7 silencing. Additionally, HSP90 is recognized as a client protein of PUS7, which is correlated with increased abundance of PUS7 in CRC. The specific HSP90 inhibitor NMS-E973 demonstrated enhanced antimetastatic activity when used in conjunction with PUS7 repression. Importantly, these findings were corroborated by analyses of human CRC tissues, where the expression of PUS7 was positively correlated with the expression of HSP90 and LASP1. Furthermore, patients coexpressing HSP90, PUS7, and LASP1 presented a poorer prognosis (Song et al., 2021).

### 6.4 Breast cancer

BC ranks among the three most prevalent cancers worldwide (Harbeck and Gnant, 2017). DKC1 levels vary widely in patients with BC, with lower levels often indicating better clinical outcomes. Silencing DKC1 in MCF-7 human BC cell lines reduces telomerase activity and rRNA pseudouridylation (Montanaro et al., 2006). In addition to their role in rRNA pseudouridylation and ribosomal biogenesis, DKC1 levels are directly correlated with telomerase activity in patients with BC. Patients with low DKC1 levels exhibit decreased telomerase activity (Gu et al., 2008). Furthermore, PUS1 has been identified as a coactivator of RAR γ-mediated gene regulation in BC cells, exerting its effect through the pseudouridylation of another coactivator, the steroid receptor RNA activator. However, the specific role of PUS1 in BC requires further clarification (Zhao et al., 2004).

### 6.5 Non-small cell lung cancer

In NSCLC, DKC1 plays a crucial role in stabilizing TERC, as indicated by the significantly decreased survival rate of patients with NSCLC with high DKC1 expression in the absence of TERC gene amplification (Penzo et al., 2015). In addition, in NSCLC, the lncRNA PCAT1 is significantly upregulated and interacts with DKC1. PCAT1 regulates the proliferation, invasion, and apoptosis of NSCLC cells through the VEGF/AKT/Bcl-2/Caspase9 pathway (Liu et al., 2021). Furthermore, a study revealed that the genetic variant rs9309336 may interfere with PUS10 expression, reducing tumor cell sensitivity to tumor necrosis factor-related apoptosis-inducing ligand (TRAIL). This genetic alteration promotes tumor cell immortalization and cancer development (Ji et al., 2020).

Several studies have demonstrated that non-small cell lung cancer (NSCLC) cell lines and tissues exhibit elevated levels of PUS7. Furthermore, PUS7 has been shown to influence the proliferation, migration, and invasion of cancer cells but has no significant effect on apoptosis. Research indicates that NSCLC patients with high PUS7 expression in tumor tissues experience a markedly reduced survival rate. These findings suggest that PUS7 may serve as a valuable biomarker for assessing postoperative prognosis in patients with non-small cell lung cancer and indicate that PUS7 could be considered an independent prognostic indicator (Zhang G. et al., 2023).

### 6.6 Prostate cancer

PC significantly contributes to male mortality worldwide and represents one of the most prevalent malignancies in men (Sekhoacha et al., 2022). Recent studies have indicated an elevated level of ψ in PC samples, suggesting its potential as a biomarker for disease progression (Stockert et al., 2019). In addition, DKC1 is markedly upregulated in patients with PC, particularly in cases of high grade and recurrence, and is associated with the expression of hTR and MK167 (Sieron et al., 2009). Deletion of DKC1 in PC cells has been shown to decrease cell proliferation, although it does not affect cell apoptosis or senescence (Sieron et al., 2009). Moreover, PUS10 has been identified as a mediator that induces TRAIL to impact the apoptosis of PC cells through a tumor necrosis factor-related mechanism. However, the precise role of PUS10 in PC requires further analysis (Jana et al., 2017).

### 6.7 Glioblastoma

GBM is the most prevalent and aggressive malignant brain tumor in adults (Davis, 2016). In GBM, PUS7 expression is typically elevated compared with that in normal brain tissue, and this high expression correlates with poor survival rates for patients, indicating a worse prognosis with higher PUS7 expression. PUS7 plays a role in controlling the tumorigenesis of glioblastoma stem cells (GSCs) by regulating codon-specific translation control of key GSC regulators through tRNA pseudouridylation. PUS7 overexpression promotes tRNA pseudouridylation, promoting tumor cell growth and self-renewal (Cui et al., 2021). Research has demonstrated that the MYC family of oncoproteins, specifically MYC and MYCN, transcriptionally activates a PUS7-dependent mRNA pseudouridylation program. This program plays a crucial role in sustaining cancer cell proliferation and tumorigenesis by enhancing ATF4-mediated metabolic reprogramming and facilitating adaptive responses that help mitigate the cellular stresses linked to increased cell proliferation and biomass production (Ding et al., 2024). In addition, DKC1 is significantly overexpressed in patients with GBM. DKC1 knockdown inhibits GBM cell proliferation, induces G1 cell cycle arrest, and reduces migration and invasion. This effect is mediated by increased expression of the cell cycle regulators CDK2 (cyclin-dependent kinase 2) and cyclin E and decreased expression of N-cadherin, MMP-2, and HIF-1α (Figure 3D) (Miao et al., 2019).

### 6.8 Ovarian cancer

OC is a significant contributor to cancer-related deaths in women, with approximately 140,000 women worldwide succumbing to OC annually (Penny, 2020). Elevated PUS7 has been observed in OC, suggesting its potential utility as a diagnostic marker and therapeutic target for this disease (Li et al., 2021). High levels of PUS7 can lead to the pseudouridylation of RAP1B mRNA by DKC1, resulting in increased RAP1B protein levels. This, in turn, promotes the proliferation and migration of tumor cells (Li et al., 2021).

### 6.9 Oral squamous cell carcinomas

OSCC is the most prevalent malignant tumor among oral cancers (Chamoli et al., 2021). In OSCC patients, DKC1 is often overexpressed in immortalized and transformed oral keratinocytes compared with primary cells, which is correlated with a greater cell proliferation rate (Alawi et al., 2010). Targeting DKC1 with small-molecule inhibitors holds promise as a potential therapy for OSCC. Moreover, saliva metabonomics analysis revealed significant upregulation of Ψ in patients with OSCC and oral leukoplakia compared with normal controls. These findings suggest that Ψ may serve as a biomarker for OSCC and oral leukoplakia (Sridharan et al., 2019).

### 6.10 Acute myeloid leukemia

Acute myeloid leukemia (AML) is characterized by the malignant transformation of myeloid stem cell precursors, affecting red blood cells, platelets, and white blood cells in addition to B and T cells (Pelcovits and Niroula, 2013). PUS7, which is enriched in stem cells, has been found to bind to various tRNA molecules, regulating the biogenesis of specific 5′tRFs containing terminal oligoguanine motifs (mTOGs). This regulation inhibits translation and is crucial for stem cell function. Deletion of PUS7 has been linked to abnormalities on chromosome 7 in myelodysplastic syndrome (MDS), which are clonal disorders of hematopoietic stem cells and progenitor cells (HSPCs) associated with a high risk of AML. Dysfunction of PUS7 and mTOGs can increase protein synthesis, impair stem cell differentiation, and contribute to leukemia development (Sperling et al., 2016). Notably, PUS7 and mTOG levels are significantly lower in HSPCs from newly diagnosed patients with MDS than in those from healthy controls, leading to higher protein production rates (Guzzi et al., 2018).

### 6.11 Malignant pituitary tumors

PAs are common intracranial tumors that are typically benign with low malignancy (Melmed, 2015). Dysfunction of DKC1 downregulates the translation of specific mRNAs containing IRES elements, including the tumor suppressor p27. A novel mutation (DKC1S485G) in DKC1 found in human pituitary adenomas has been shown to significantly impact DKC1 stability and pseudouridylation activity, leading to decreased p27 protein levels (Bellodi et al., 2010). A study in mice heterozygous for p27 and with DKC1 mutations demonstrated that reduced DKC1 levels decrease p27 IRES-mediated pituitary translation, consequently increasing spontaneous pituitary tumorigenesis. These findings suggest a genetic interaction between DKC1 and p27 in pituitary tumorigenesis (Bellodi et al., 2010).

## 7 Conclusion

This review provides an overview of the chemical structure, synthesis, and regulation of Ψ in RNA. Initially, the chemical structure and enzymatic mechanism of pseudouridylation were reviewed. Subsequently, the effects of pseudouridylation on various RNA molecules were briefly introduced, along with methods for detecting Ψs. This review focused on the impact of RNA pseudouridylation on different cancers. While the biochemical and structural effects of Ψ in various biological systems have been investigated, its precise biological function remains elusive and warrants further exploration. Dysregulation of Ψ in the transcriptome is implicated in several human diseases, leading to alterations in RNA metabolism. Current research suggests that “writer” regulators, mainly DKC1 and PUS enzymes, may help regulate cancer onset and progression, making them potential therapeutic targets for cancer treatment and prognosis. Future advances in RNA pseudouridylation will largely hinge on progress in sequencing technology. An essential task in the field is to identify Ψ “reader and eraser” proteins and assess their suitability as therapeutic targets for cancer and other diseases. Several questions regarding the crosstalk between Ψ and other RNA modifications remain unanswered. While numerous studies on single RNA modifications have demonstrated their impact on diseases, it remains uncertain whether the relationships and actions between Ψs and other types of RNA modifications differ within specific diseases. In addition, whether future targeted drugs are influenced by other modification types, potentially resulting in unforeseen crosstalk, requires further investigation.

## Glossary

Ψ: Pseudouridine
mRNA: Messenger RNA
tRNA: Transfer RNA
tsRNA: tRNA-derived small RNA
rRNA: Ribosomal RNA
snRNA: Small nuclear RNA
lncRNA: Long non-coding RNA
HCC: Hepatocellular Carcinoma
GC: Gastric Cancer
CRC: Colorectal Cancer
BC: Breast Cancer
NSCLC: Non-Small Cell Lung Cancer
PC: Prostate Cancer
GBM: Glioblastoma
OC: Ovarian Cancer
OSCC: Oral Squamous Cell Carcinoma
AML: Acute myeloid leukemia
MDS: Myelodysplastic syndrome
HSPCs: Hematopoietic stem cells and progenitor cells
PUS: Pseudouridine Synthase
U: Uridine
RNP: Ribonucleoprotein
DKC1: Dyskerin Pseudouridine Synthase 1
PUS1: Pseudouridylate Synthase 1
PUS7: Pseudouridylate Synthase 7
PUS10: Pseudouridylate Synthase 10
pre-mRNA: Premessenger RNA
tRF: tRNA-derived Fragment
snRNP: Small Nuclear Ribonucleoprotein
TK6 cells: Human lymphoblast cell from spleen, Epstein-Barr virus transformed
HEK293T cells: Human Embryonic Kidney cells 293
PSI seq: Pseudouridine Site Identification sequencing
CeU seq: N3-CMC-enriched Pseudouridine sequencing
CMC: N-cycloxyl-N'-(2-morpholinoethyl)-carboximide
RBS seq: RNA Bisulfite Sequencing
BID-seq: Base-by-Base Identification sequencing
RT-PCR: Reverse Transcription-Polymerase Chain Reaction
CLAP: CMC-RT and Linker-Assisted PCR
TERC: Telomerase RNA Component
MYC genes: Encoding the c-MYC protein
MKI67 genes: Encoding the Ki-67 protein
TNM: Primary tumor local lymph node distant metastasis staging
TRAIL: Tumor necrosis factor-related apoptosis-inducing ligand
Rap-1b: Ras-related protein 1b
IRES: Internal Ribosomal Entry Site.
